# Direct Ink Writing Additive Manufacturing of Polyimide Aerogels

**DOI:** 10.3390/gels11120940

**Published:** 2025-11-23

**Authors:** Bo Chen, Qiyang Jiang, Jianhu Jiang

**Affiliations:** 1 Yangtze Delta Region Institute (Huzhou), University of Electronic Science and Technology of China, Huzhou 313001, China; chenbo@csj.uestc.edu.cn; 2 Agriculture Ministry Key Laboratory of Healthy Freshwater Aquaculture/Key Laboratory of Freshwater Aquatic Animal Genetic and Breeding of Zhejiang Province, Zhejiang Institute of Freshwater Fisheries, Huzhou 313001, China

**Keywords:** polyimide aerogels, aerogel properties, direct ink writing, aerogel ink preparation, application

## Abstract

Polyimide aerogels (PAs) are ideal for applications in thermal protection, lightweight electronics, and energy devices due to their excellent mechanical properties, ultra-low density, extremely low thermal conductivity, and high thermal-oxidative stability. Conventional PA manufacturing involves a sol–gel process followed by post-processing (drying and imidization). However, PAs fabricated using this method are geometrically limited by the mold shape and are fragile, have poor sample machinability, and are prone to shrinkage and deformation. Direct ink writing (DIW) additive manufacturing (AM) overcomes these limitations of conventional manufacturing processes by extruding ink to construct architectural lattices with high dimensional fidelity, enabling the fabrication of complex, conformal, and multi-scale structures. DIW AM can produce PA components that are thermally and electrically stable, as well as geometric freedom, thus supporting high-precision and functional hierarchical design. This review provides the first overview of DIW AM of PAs. By summarizing printable ink formulations, printing parameters, drying routes and thermal/chemical imidization processes, as well as applications of printed samples, it comprehensively describes the current state of the art in DIW additive manufacturing of PAs and highlights key technical bottlenecks (printability vs. porosity trade-off, economic and environmental, etc.). It also outlines possible future research directions.

## 1. Introduction

Aerogels are a class of materials with high porosity, extremely low apparent density and thermal conductivity [1,2,3]. These materials contain a tunable nanostructured network consisting of nanoparticles, nanofibers, or nanosheets, with pores ranging in size from the mesoscopic to the macroscopic, and a porosity typically exceeding 90% [4,5,6,7,8,9]. Therefore, aerogels can achieve a variety of extraordinary physical properties, including ultralow density, high specific surface area, low thermal conductivity, and excellent thermo-oxidative stability [10,11,12,13]. This makes them ideal for use in a range of applications in fields such as aerospace, thermal management, insulation materials, sensors, shock absorption technology, biomedicine, healthcare, energy storage, artificial intelligence, and other related fields [14,15,16,17,18,19,20,21,22,23,24,25,26].

Since the discovery of silica aerogel (SA) by Samuel Kistler in 1931, inorganic (oxide) aerogels, polymer aerogels (such as resorcinol-formaldehyde, polyurea, and polyimide (PI)), carbon aerogels (via carbonization), bio-derived aerogels (such as cellulose and chitosan), and composite aerogels integrating multiple phases have also been discovered [1,27,28,29,30,31,32,33,34,35,36,37]. PAs, as polymer aerogels, possess low density and low thermal conductivity [38]. Their imide backbone overcomes the limitations of organic aerogels in terms of low thermal stability at high temperatures, resulting in excellent thermo-oxidative stability, mechanical elasticity, and low dielectric loss. Compared to ceramic aerogels, organic aerogels have relatively low thermal stability. Many organic aerogels begin to oxidize in air at temperatures above ~300–400 °C. In contrast, inorganic aerogels (e.g., silica and alumina) can withstand higher temperatures. Fully aromatic polyimides extend the usable temperature range to ~400–500 °C in air [19,39,40,41,42,43,44,45,46,47]. However, PAs are susceptible to severe volume shrinkage and structural collapse at temperatures between 200 and 400 °C [39]. Filling polyimide aerogels with inorganic materials, such as silica and magnesia, helps achieve shape retention and dimensional stability at moderately high temperatures [48,49,50].

PAs are typically prepared using a sol–gel route, using poly(amic acid) (PAA) or poly(amic acid) ammonium salt (PAAS) precursors, followed by solvent exchange/aging to enhance the wet network, solvent removal via supercritical carbon dioxide (CO_2_) drying or freeze-drying, and final imidization (thermal or chemical) [51]. This process is limited by the mold geometry and is costly when preparing products with complex three-dimensional structures [52]. The resulting PAs suffer from poor shape retention and limited dimensional and shape customization in practical applications [53]. With the development of additive manufacturing (AM), it has become an effective approach to overcoming the limitations of traditional aerogel molding processes [54]. AM technology maintains the porous microstructure of PAs while also enabling the design of their macroscopic spatial structures, making PAs competitive in practical applications such as aerospace and thermal management [55].

AM uses a bottom-up additive strategy, aided by modeling software, to precisely design the required dimensions and complex three-dimensional structures [56]. This simplifies the process cycle and reduces material consumption [57]. Typically, the aerogel AM process involves four steps: (i) development of a printable ink formulation, (ii) design and 3D printing of customized geometries, (iii) an optional post-curing step for solidification or chemical modification, (iv) selection of appropriate drying techniques to maintain shape fidelity and pore structure [58,59,60,61,62]. Research on 3D-printed aerogel scaffolds was first reported in 2015, marking the first application of AM technology combined with aerogels [63]. Since then, AM has flourished. Direct ink writing (DIW) technology is a pressure-driven, viscoelastic, shear-thinning ink extrusion technique [64]. As a form of AM, this technology utilizes a three-axis motion platform for ease of operation and can produce the desired ink from a wide range of raw materials (aromatic dianhydrides, diamines, oligomeric PAA, fillers such as silica, cellulose nanofibrils, carbon nanotubes) have been employed to tune mechanical, thermal and dielectric properties [65,66,67,68,69,70,71,72]. It offers excellent aerogel moldability and low cost, which make it as a widely applicable preparation method for liquid-solid sol–gel systems. In 2021, composite PAs were first 3D printed using DIW [73]. The realization of PA aerogel DIW printing relies heavily on the development of inks, where the rheological properties of the ink are crucial parameters for the precision and shape retention of printable structures [74]. Sufficiently high ink viscosity allows for the self-support of complex three-dimensional structures [75,76]. The ink’s shear-thinning behavior ensures continuous extrusion during the printing process [77,78,79]. The ink’s storage modulus G′ and loss modulus G″ ensure that the ink quickly recovers its gel properties after extrusion from the nozzle, resulting in optimal 3D printed structures [80,81,82]. To overcome the rheological challenges of DIW printing inks, various fillers have been incorporated into the sol. Some groups incorporate inorganic nanoparticles (e.g., silica, alumina) or nanofibers to enhance mechanical strength, while others use organic rheology modifiers (cellulose derivatives, graphene) to improve ink printability [39,83,84,85,86,87]. These inorganic fillers improve the ink’s rheological properties and further enhance the polyimide aerogel’s flame retardancy, thermal insulation, and other properties [88].

Based on the current state of additive manufacturing of PAs using DIW, this review summarizes the latest progress and applications in DIW-printed PAs and offers an outlook for future development. The development of inks, 3D printing processes, drying, thermal treatment and potential application fields for existing DIW-printed PAs are summarized. Finally, the key challenges of current DIW-printed PAs are reviewed to understand potential avenues for upcoming innovations.

## 2. DIW 3D Printing of PAs

While this review focuses on direct ink writing (DIW), several other additive-manufacturing (AM) strategies have been applied or considered for aerogel fabrication, a brief comparison clarifies the relative strengths and limitations.

Direct cryo-writing (DCW) extrudes viscous inks directly onto a cryogenic substrate or into a cold environment so filaments freeze quasi-instantly [89]. This approach eliminates the need for immediate gelation and enables retention of macroporous architectures without rapid chemical curing [90,91,92]. DCW is particularly useful for solvent-rich systems that can be frozen and subsequently sublimed (freeze-drying) [93,94]. Limitations include the need for cryogenic hardware, lower dimensional precision due to thermal contraction during freeze–thaw, and restricted scalability in continuous manufacturing lines [95].

Inkjet deposits discrete droplets by piezoelectric or thermal actuation and can realize very high lateral resolution for thin films or small features [96]. Inkjet suitability requires low-viscosity inks (typically <50 mPa·s), which is challenging for aerogel precursors that rely on high solids or yield stress for filament support [97,98]. Inkjet is more appropriate for patterned coatings or hybrid processes (e.g., printing functional inks onto preformed aerogel scaffolds) rather than building freestanding aerogel lattices [99,100,101].

Vat polymerization (SLA/DLP) cures photosensitive resins layer-by-layer using point (SLA) or projected (DLP) light [102,103,104]. It yields excellent resolution and surface finish but requires photopolymerizable precursors that are generally incompatible with high-temperature polyimide chemistry [105,106]. Hybrid strategies that combine photo-curable binders with sacrificial templates have been explored for carbon/aerogel precursors, but the need for post-curing and the limited library of high-temperature resins limit direct applicability to PAs [107,108,109].

DIW accepts a broad viscosity window (10^2^–10^6^ mPa s), works with solvent-based PAA and PAAS chemistries commonly used for polyimide aerogels, and supports multi-material and graded architectures at moderate resolution (typical filament/feature sizes 100–1000 µm) [59,110]. DIW’s principal challenges are rheology engineering (printability vs. porosity trade-offs) and post-processing (drying and imidization) to preserve geometry and nanoscale porosity [111,112].

### 2.1. Preparation and Composition of PAs Ink Formulations

DIW formulations reported to date employ either aqueous dispersions of PAA (e.g., pyromellitic dianhydride-4,4-oxydianiline (PMDA-ODA)) or PAA in polar aprotic solvents (e.g., DMAc, NMP), with solvent choice determined by precursor chemistry and downstream drying/imidization [73]. The ink is then subjected to a solidus window in which it exhibits pronounced shear-thinning behavior, with a yield stress exceeding gravity and capillary loads within the intended filament span and rapid recovery of thixotropy after shear cessation [113]. In additive-free, time-assisted processes, mild pre-aging and temperature control can promote sol–gelation without the addition of fillers [74]. During the sol–gel process, rapidly gelling sols is desirable for achieving printability within a short time. However, the associated rapid gelation kinetics may cause nozzle clogging due to solidification of the ink [114]. Conversely, slower-gelling sols to prolong the time window for ink loading could also increase processing time, especially for large-scale structures [115]. Therefore, optimizing the rheology properties of inks is essential to achieve both printability and structural integrity without compromising processing efficiency. More recently, progress has been reported with the introduction of various rheology-modifying additives into the sol, such as cellulose nanocrystals (CNC), bacterial cellulose (BC), fumed silica, and silica aerogel particle (SAP) [116,117,118,119]. These additives not only improve the ink’s rheological properties but also modify the PA’s porosity, density, and optical clarity [87]. Table 1 shows examples of PA inks suitable for DIW 3D printing. The introduction of hydrophilic SAP as rheology modifiers improves the printability and shape retention of 3D-printed composite aerogels [51]. SAP forms a hydrogen-bonded network with PAA, acting as a nanoporous scatterer within the aerogel, reducing thermal conductivity while enhancing dielectric properties, hydrophobicity, and flame retardancy [84]. Fibrous modifiers BC/CNC form a percolating network, increasing the G′ and yield stress (τ_y_) at moderate loading and suppressing drying shrinkage, thereby improving dimensional fidelity for highly stacked structures and overhang-sensitive toolpaths [120]. Multifunctional additives can improve the rheological properties of inks while also enhancing the properties of printed structures. Common composite ingredients include BC (0.5–5 wt%), CNCs (0.1–2 wt%), graphene/Carbon nanotubes (CNTs) (0.1–1 wt%), and silica nanoparticles (1–20 wt%), used, respectively, for mechanical reinforcement, rheology augmentation, conductivity, and pore templating [121,122,123,124,125,126,127,128]. For example, halloysite nanotubes (HNTs) act as a hollow tube scaffold which can enhance toughness, Fe^3+^ accelerates gelation through coordination and a graphene oxide/reduced graphene oxide (GO/rGO) surface layer enables photothermal harvesting or electromagnetic interference (EMl) shielding without compromising DIW processability [129,130,131]. In other works, Composite aerogels containing fillers have also been reported to improve the yield stress and microstructural stability of the extruded filaments [132]. However, the addition of inorganic fillers increases aerogel density and thermal conductivity; thus, adjusting ink composition must be carefully balanced against the requirements of the target application [133].

### 2.2. Requirements for the Rheological Properties of Inks

The flow and transfer of ink are fundamental to forming a self-supporting layer in DIW [137]. Ink printability refers to the ability of the ink to be extruded smoothly through a specific nozzle as a continuous filament, allowing for the fabrication of precise structures without particle buildup and nozzle clogging [132]. Shape fidelity refers to the ink’s ability to maintain its printed shape after deposition, drying, and post-processing [138]. Therefore, the ink’s rheological properties are crucial parameters determining its printability and shape fidelity (Table 2). Printable inks generally exhibit shear thinning properties. Shear thinning is a non-Newtonian property where viscosity decreases with increasing shear rate [139]. Viscosity, defined as the ratio of shear stress to shear rate, exhibits a linear or nonlinear relationship with increasing shear rate [140]. This property facilitates extrusion of the ink at lower pressures, thus ensuring extrudability. The viscosity of ink depends on factors such as formulation composition and temperature [111]. Typically, DIW inks have viscosities ranging from 0.1 to 1000 Pa s at shear rates of approximately 0.1 s^−1^ [141].

**Figure 1:**
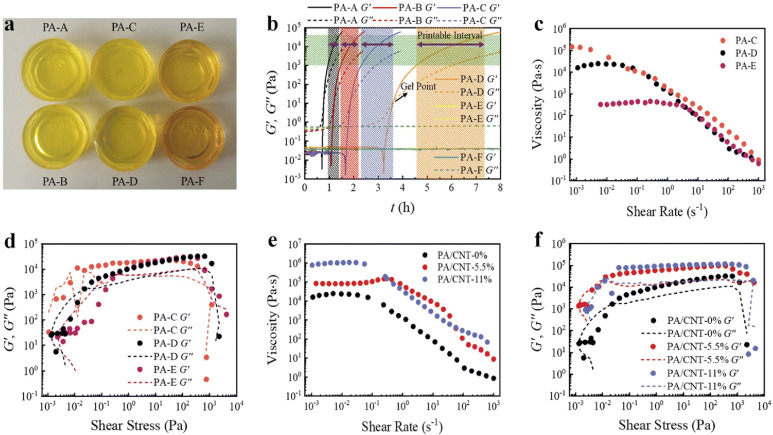
(**a**) Image of PAs inks. (**b**) G′ and G′′ of PAs inks. (**c**) Viscosity of different PAs inks. (**d**) G′ and G′′ of PAs inks. (**e**) Viscosity of different PA/CNT inks with. (**f**) G′ and G′′ of PA/CNT inks.

Yang et al. prepared PA (prepared by DMBZ, BTDA, TAB, NMP, AA, PD, CNTs, aqueous ammonia and ethanol absolute) inks with different PD to BTDA molar ratios (Figure 1a). The continuous evolution of different inks G′ and G″ over time (Figure 1b). Figure 1c,e shows all inks (PA inks and PA/CNTs inks) presented exhibit shear-thinning behavior (viscosity decreases with increasing shear rate), a characteristic that facilitates extrusion through a nozzle under reasonable applied pressure. Shear thinning governs the in-nozzle flow regime, by contrast, post-deposition filament stability relies on rapid thixotropic recovery so that the storage modulus (G′) becomes dominant at low shear. Immediately after deposition we target G′ > G″ (storage modulus greater than loss modulus) to resist filament sagging and preserve printed geometry. By convention, a viscoelastic “sol” shows G″ > G′ at small strains whereas a “gel” exhibits G′ > G″; printable DIW inks combine shear thinning with fast recovery to a gel-like state after extrusion. Figure 1d demonstrates the printability of the pure PA ink, where the G″ is greater than the G′ when the shear stress exceeds the yield stress of the ink. Reported practical windows for DIW PA inks are zero-shear viscosities ~10^2^–10^6^ mPa·s (depending on solids content and additives), yield stresses in the ~10–10^3^ Pa range for self-support, and recovery half-times on the order of 1–10 s for good stacking fidelity. Furthermore, the ink should exhibit a well-defined linear viscoelastic region (LVR), where G′ remains constant at low shear stresses (Figure 1f) [142].

**Figure 2:**
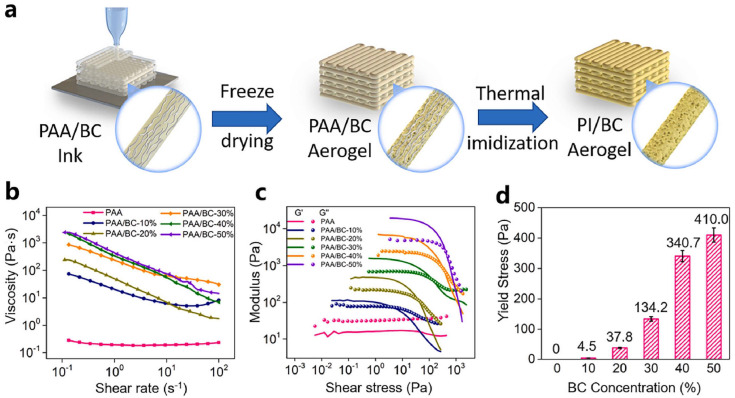
(**a**) DIW 3D printing PI/BC composite aerogels. (**b**) Steady-state shear viscosity of the PAA/BC inks. (**c**) G′ and G″ of PAA/BC inks as a function of shear stress. (**d**) The yield stress of PAA/BC inks with different contents of BC.

As shown in Figure 2a, Ma et al. also showed similar results when preparing inks by combining BC with PAA (Prepared by mixing BAPP, dimethyl sulfoxide (DMSO) and BPADA). Pure PAA solutions exhibited shear-thinning behavior at low shear rates, where the entanglement of the PAA polymer chains was broken during shear. With the addition of BC, an order of magnitude increase in apparent viscosity was observed, and the ink exhibited shear-thinning behavior (Figure 2b). The observed increase in viscosity was attributed to the breaking of the original intramolecular hydrogen bonds and the formation of new intermolecular hydrogen bonds between PAA and BC. Furthermore, Figure 2c shows the modulus of the PAA/BC ink gradually decreases with increasing shear stress, due to the breakdown of the PAA/BC network. Figure 2d shows the progressive dominance of viscous behavior when shear stress exceeds the yield stress, consistent with shear-induced network breakdown during extrusion, followed by modulus recovery after shear cessation [134]. A higher yield stress indicates a greater resistance to deformation after extrusion, allowing the ink to better maintain its original structure and effectively improving the stability of the printed structure [52].

### 2.3. Ink Preparation Processes for Printed PAs

DIW 3D printing technology for preparing PAs typically involves a prepared printable ink, followed by drying through methods such as supercritical drying and freeze drying [74]. Post-drying sometimes requires ball milling, imidization, heating, or UV irradiation to ultimately obtain PAs with a complete structure and the desired functionality [143]. However, the thermal imidization process in aqueous PAA systems and organic solvent-based systems affects printing fidelity and the printable time window [135]. Therefore, silica aerogel particles can be used as fillers to modulate inks and enhance their properties.

**Figure 3:**
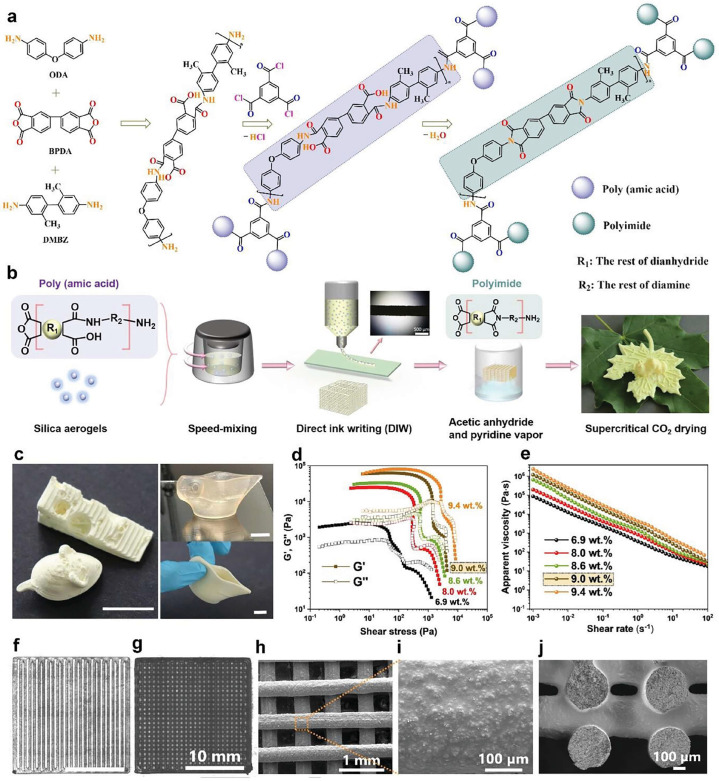
(**a**) Synthesis route of PI. (**b**) DIW of PI-silica composite aerogels from a PAA-silica aerogel ink. (**c**) Printed objects (scale bar is 10 mm). (**d**) The relationship between G′ and G″ as a function of shear stress. (**e**) Steady-shear rheological measurements were taken for inks containing different amounts of silica aerogel filler (6.9%, 8.0%, 8.6%, 9.0% and 9.4% by weight). (**f**) image of a printed one-layer grid. (**g**) image of a printed five- layer grid. (**h**) SEM image of dried grid and filaments. (**i**) SEM image of the surface of dried filament. (**j**) SEM image of the cross-section of the dried grids.

Wu et al. incorporated 4–20 μm silica aerogel particles into PAA (prepared by BPDA, ODA, DMBZ), the synthesis of PAs as shown in Figure 3a. In solution, ink flowability is improved and the performance of the composite aerogel is enhanced. Figure 3b shows the preparation process. Based on vapor-phase induced curing and chemical imidization with acetic anhydride (AA) and PD vapor, post-printing curing can be achieved to extend printing time. The toxic DMAc solvent is exchanged for ethanol through imidization gelation. After supercritical CO_2_ drying, the printed structure maintains good shape with a homogeneous dispersion of silica aerogel particles. Figure 3c–e demonstrate the printability of the ink. However, the printing precision of this method is only 410 μm, which still leaves significant room for improvement. Figure 3f–j show the morphology after printing [135].

**Figure 4:**
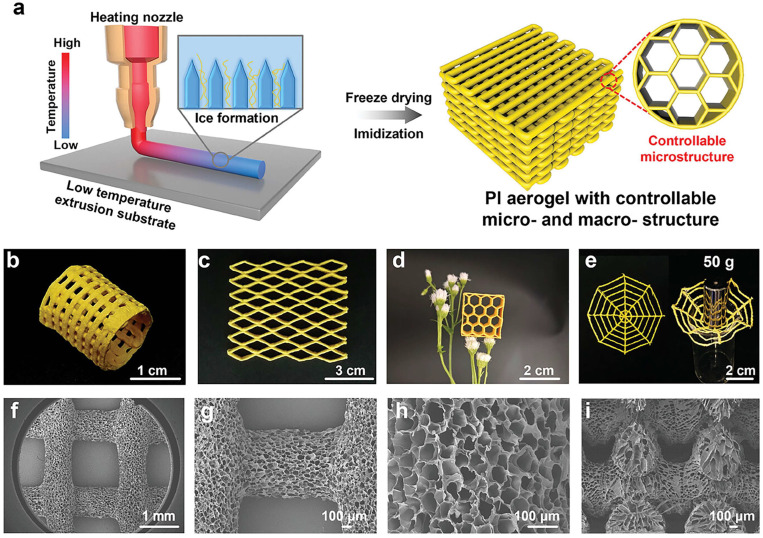
Schematic and structure of FADIW PAs. (**a**) Schematic diagram of 3D printed PAs by FADIW technology. (**b**,**c**) FADIW PAs with different structures. (**d**) FADIW PAs honeycomb with low density (0.04 g cm^−3^) that can be placed on flower. (**e**) FADIW PAs with spider web structure, which can withstand 50 g weights. (**f**–**h**) SEM images of the top view of the 3D printed PAs. (**i**) Cross-sectional SEM images of the 3D printed PAs.

Xue et al. printed a PAA printable ink using a nozzle with a diameter of 400 μm (Schematic and structure as shown in Figure 4a). After heating the syringe to >20 °C, they successfully extruded filaments onto a temperature-controlled substrate between −60 °C and 10 °C. This cryo-assisted method is also applicable to inks containing fillers such as PI/HNTs or PI/rGO. After subsequent freeze drying and thermal imidization, 3D-printed PAs are obtained, as showed in Figure 4b–e. However, the cryo-assisted method requires precise control of temperature and has limited precision (the morphology after printing as shown in Figure 4f–i).

**Figure 5:**
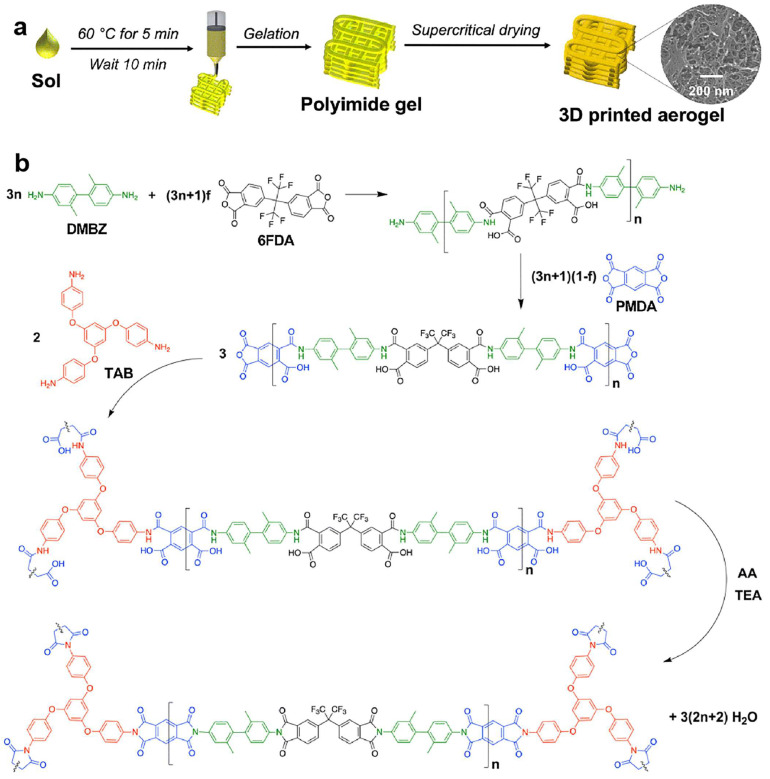
(**a**) DIW 3D printing PAs. Mild heating of the sol speeds gelation to produce a 3D printable ink, as defined by rheological properties. (**b**) Synthetic scheme of polyimide aerogels used here (f = 0.25 and *n* = 60).

Ciera E. Cipriani et al. prepared a printable ink using a mild heat treatment (Figure 5). The printable ink was extruded through a nozzle to print the designed structure, which was then placed in a Parafilm-sealed container to prevent solvent evaporation. Subsequently, supercritical drying was performed at 25 °C and 7 MPa. The resulting polyimide aerogel retains the characteristic microstructure, mechanical properties, and thermal behavior of the traditionally cast material. This method is simple, cost-effective, and requires no particulate fillers, extreme conditions (e.g., sustained operation at 400–600 °C in air, exposure to concentrated salts or acidic gases, or cryogenic cycling to <−150 °C), or additional post-processing steps [74]. However, the fidelity of the structures printed using this method is limited, and controllable shrinkage can be challenging.

## 3. Applications of DIW 3D-Printed PAs

PAs, due to their exceptional thermal, mechanical, chemical, and electrical properties, as well as their structural morphology, are ideal for applications in thermal insulation, aviation, energy harvesting, air filtration, microelectronics, and environmental remediation [144]. Although conventionally fabricated PAs hold considerable promise for future applications, their advancement is hindered by the difficulty in producing high-precision, complex architectures [145]. DIW 3D printing, demonstrating superior micron-scale processing capabilities and design flexibility, addresses the limitations of low precision and simple structures, expanding the application boundaries of PAs [73]. This section summarizes the existing literature, focusing on PAs fabricated using DIW AM for applications in thermal management, energy storage, electronics, biomedicine, and light harvesting.

### 3.1. Thermal Management

Heat is primarily transferred through three heat conduction mechanisms: solid-phase conduction, gas-phase conduction, and radiation conduction [146]. Aerogels generally possess low solid-phase thermal conductivity, large specific surface area, and long solid-phase conduction paths [146]. Therefore, radiation heat transfer in aerogels is negligible at room temperature, and the main heat conduction mechanism is gas-phase heat transfer at room temperature [147]. PAs can achieve a heat transfer efficiency as low as 0.014 W m^−1^K^−1^ [148]. Due to their nanoporous network structure, ultra-low thermal conductivity, and high porosity, they are considered one of the best thermal insulation materials [149]. Therefore, thermal management is the most widely used application area for PAs. However, in thermal insulation applications that demanding high precision and complex structures, the desired properties (e.g., precision assembly) for PAs are difficult to achieve through traditional manufacturing processes [53]. Precision assembly is required to fabricate desired shapes, minimize heat transfer, and achieve effective insulation.

**Figure 6:**
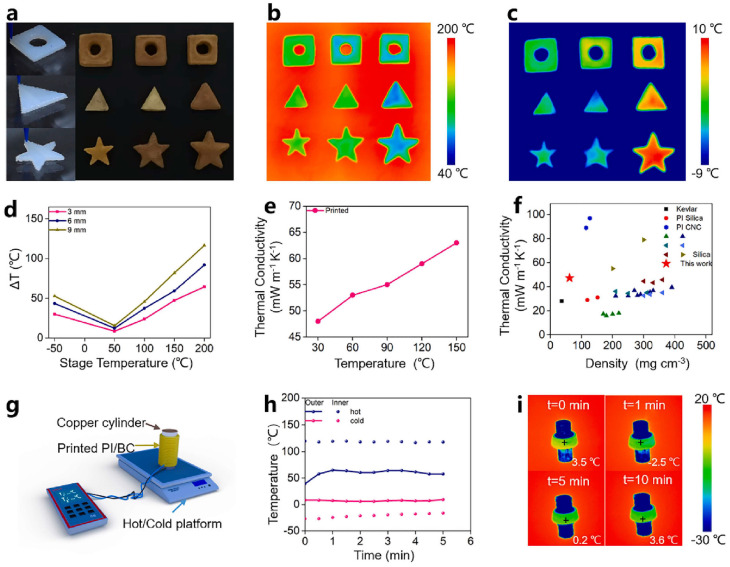
Fabrication and assessment of 3D-printed PI/BC aerogels for thermal management. (**a**) Demonstrates the printability of various structures and the resulting aerogels with different heights. The thermal insulation performance is visualized via infrared imaging on a 200 °C hot plate (**b**) and a −30 °C freezing plate (**c**). (**d**) Quantification of the insulation efficacy as the absolute temperature difference (|ΔT|) across aerogels of different heights. (**e**) The thermal conductivity of the aerogel is plotted against temperature, and (**f**) its overall thermophysical properties are benchmarked against other printed aerogels. The application as a protective coating is shown in (**g**), where a schematic depicts an aerogel ring on a copper rod under thermal stress. (**h**) The corresponding temperature profile across the coating is recorded, and (**i**) the thermal stability is further demonstrated by infrared imaging over 10 min on a freezing substrate.

Ma et al. combined BC with a PAA solution to create a printable ink. The water-soluble PAA was obtained by polycondensation of PMDA-ODA with the addition of TEA. Figure 6a shows printed triangular/pentagonal/square geometries with central holes. Figure 6b,c are infrared thermal images of the printed PI/BC aerogels on a 200 °C hot plate and a −30 °C freezing plate, respectively. A series of square, triangular, and pentagonal samples with varying heights were then printed and placed on a temperature-adjustable platform for testing. Figure 6d quantifies the temperature difference |ΔT| between top and bottom faces as a function of sample height. Figure 6e reports thermal conductivity vs. temperature for PI/BC aerogels. Its thermal conductivity is as low as 47.4 mW m^−1^K^−1^ at 30 °C and 61.8 mW m^−1^K^−1^ at 150 °C. Figure 6f compares conductivity and density across reported printed aerogels. Figure 6g shows the thermal insulation performance test of PAs. PAs rings were printed on the surface of a copper column, and the temperature inside and outside the aerogel rings was recorded after the copper column was heated. A 1 cm thick insulating tube or pipe is wrapped around a copper block and placed upright on an adjustable platform. The copper rod is kept at 120 °C, while the PA surface temperature is maintained at around 56 °C, resulting in a temperature difference of up to 64 °C. When the copper rod was at −30 °C, the surface temperature of the PAs remained above 0 °C (Figure 6h), demonstrating the excellent thermal insulation capabilities of DIW-printed PAs. The PAs surface temperatures on the heated and unheated copper rods were recorded by infrared thermography after 10 min (Figure 6i). Infrared thermal imaging showed that the temperature difference between the top and bottom surfaces of the printed aerogel increased with its thickness. After the bottom temperature reaches 200 °C, the surface temperature remains below 90 °C. The bottom temperature is around −50 °C and the surface temperature remains above 10 °C [134].

**Figure 7:**
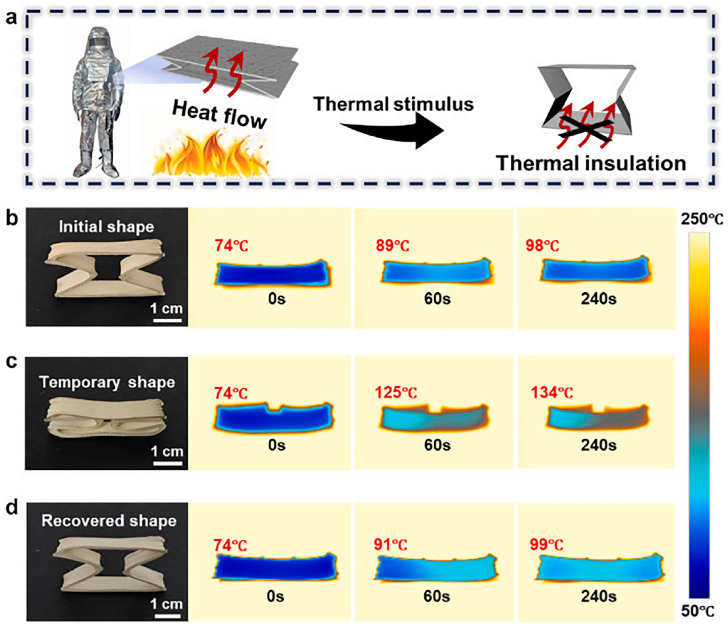
(**a**) Design of an intelligent switchable thermal insulation composite aerogel. The thermal insulation performance of the 3D-printed PI/SAP composite aerogel is shown in its (**b**) original, (**c**) temporarily programmed, and (**d**) recovered states.

Fu et al. successfully fabricated PAs with flame-retardant and thermal-insulating properties by incorporating SAP into a PAA precursor. Combustion tests demonstrated that the SAP-incorporated PA exhibited excellent flame retardancy, with merely slight reddening and slow carbonization observed. After removal from the flame, no smoke or melting occurred which indicates a good dimensional stability. The incorporated SAP acts as a physical barrier in the composite aerogel, impeding the penetration of flammable molecules and oxygen, thereby preserving the internal structure. After combustion, a carbon layer forms within the aerogel’s interior, which can preserve its porous structure within the aerogel and hinder heat conduction. The PI/SAP composite aerogel expands spontaneously in its folded state, eliminating the effects of flame or high temperature, as shown in Figure 7a. In contrast, pure PAs underwent rapid and complete carbonization. After extinguishing, the total volume decreases by 70% of pure PAs. Composite systems with SAP typically show lower shrinkage ~17% (Figure 7b). The printed composite aerogel maintains a stable surface temperature of 98 °C after heating at 250 °C. After folding, it can be used as a filling in fire-resistant clothing with heat-induced deformation capabilities. The folded structure will spontaneously expand when exposed to fire or high temperature, eliminating the impact of flames and high temperatures on the human body. After compression, the composite aerogel stabilizes at 134 °C, reducing its thermal insulation capacity (Figure 7c). Once the aerogel has returned to its permanent shape, its insulating properties are restored and its surface temperature returns to 98 °C (Figure 7d). This demonstrates that the thermal conductivity varies with the intelligent switching of the aerogel structure. These SAP-combined PAs have potential applications in intelligent deformation and thermal insulation [84].

In pure PAs, the imide backbone maintains structural integrity across a wide temperature window, while composites with fillers such as SAP offer broad thermal insulation performance, including transient flame exposure. Experimental results demonstrated that at the same thickness, DIW PAs/SAP (PAA from BAPP/BPADA with SAP addition) composites exhibit enhanced temperature protection, as evidenced by a lower backside temperature rise under torch testing compared to the traditional fiber-based insulation materials [84]. In electronics, PAs can be used to protect heat-sensitive components by 3D printing conformal housings and gaskets. In aerospace, gradient panels with a sparse core and denser skin layers can effectively integrate weight reduction with thermal protection. Reliability under repeated thermal shock and oxidative aging remains a key issue for scale-up, prompting the integration of steady-state and transient heat flux protocols. Furthermore, the performance of aerogels within such systems is temperature-dependent; at medium to high temperatures, their thermal conductivity rises significantly above room-temperature levels due to enhanced infrared radiation. Li et al. reached similar conclusions regarding recyclable polyimide composite aerogels (polyimide powder mixed with the dimethylacetamide and the silica aerogel particles) [85]. Yu et al. also fabricated PI/SAP composite aerogels exhibiting excellent flame retardancy and thermal insulation properties from −50 °C to 1300 °C [51].

**Figure 8:**
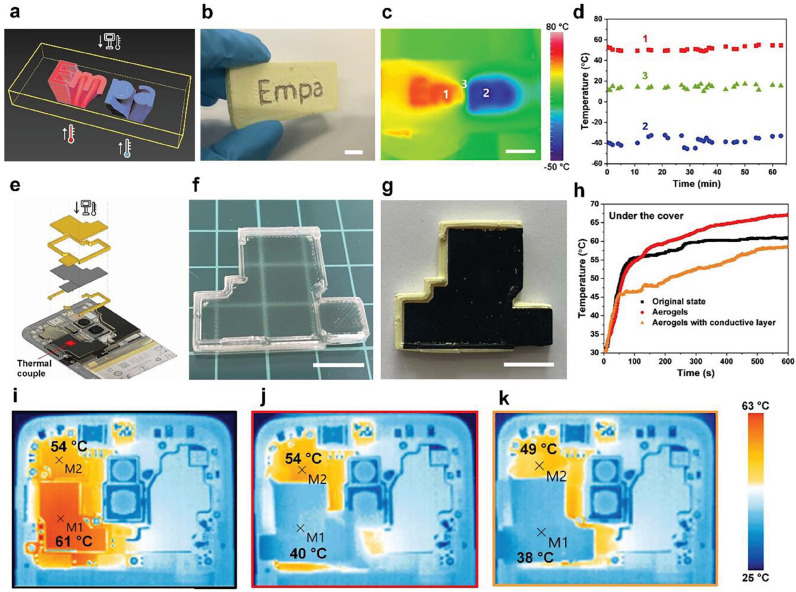
Thermal management through the integration of thermal insulator and conductor. (**a**) 3D model with spatially complex negative space. (**b**) Printed composite aerogel object where the channels are filled with conductive silver paste (scale bar is 10 mm). (**c**) Infrared image with the letters “Em” heated by a resistive heater from the bottom, and “pa” connected to a cold sink (scale bar is 10 mm). (**d**) Temperature at positions 1 to 3. (**e**) Scheme for the cellphone thermal guide cover application. The fabrication results show (**f**) the printed cover in a wet state and (**g**) the dried cover with a laser-etched graphene layer (scale bar: 10 mm). The thermal performance was characterized by measuring temperature evolution (**h**) and obtaining infrared images during operation for three configurations: (**i**) bare cellphone, (**j**) aerogel-covered, and (**k**) aerogel-covered with an etched conductive layer.

In high-power, highly integrated microelectronics such as smartphones, heat dissipation typically relies on a combination of a thermal conductor and an insulator to force heat to diffuse in two dimensions [150]. However, this design does not effectively dissipate heat within the smartphone. Wu et al. employed a single PAs structure composed of a DIW-printed thermal conductor and a thermal insulator to control and direct heat dissipation. Figure 8 illustrates the complete workflow and functional validation of DIW-printed polyimide aerogel structures for integrated thermal-management applications. The initial design model (Figure 8a) defines the architected layout of insulating regions and embedded conductive pathways, which is faithfully realized in the printed composite shown in Figure 8b. Under asymmetric heating and cooling conditions, the infrared thermal image in Figure 8c demonstrates the intended spatial modulation of surface temperature, while the corresponding temporal temperature profiles in Figure 8d confirm directional heat transport governed by the conductive channels. Building on this concept, Figure 8e–g present the design, fabrication, and post-processing of a conformal aerogel-based thermal-guidance cover for a mobile device, including the as-printed geometry and the final component functionalized with a laser-patterned graphene conductive layer. The thermal-performance comparison in Figure 8h reveals that the hybrid aerogel–conductor architecture effectively lowers the device’s peak operating temperature and enhances lateral heat spreading, a result further visualized by infrared thermal maps in Figure 8i. The completed assembly shown in Figure 8j confirms uniform coating distribution, structural fidelity, and close conformity to the device surface, collectively demonstrating the practicality and multifunctional potential of DIW-fabricated polyimide aerogel architectures for advanced thermal-management systems. The insulator is safe to touch and protects surrounding heat-sensitive components from overheating. The conductive graphene layer within the insulating shell successfully dissipates heat, reducing the internal and surface temperatures of the smartphone, rather than confining it within the insulating shell (Figure 8k) [135].

### 3.2. EMI Shielding and Dielectric Substrates

Low *k* dielectric materials enable faster signal transmission and minimized signal loss in high-frequency microelectronic devices such as 5G communication systems and radio frequency antennas [151]. Low *k* dielectric materials can significantly reduce the capacitance and resistance-capacitance delay between metal interconnects [152]. Therefore, polymers with ultra-low dielectric constants (*k*) are a key cornerstone for the rapid development of the electronics and communications industries. PAs, due to their high porosity and inherent polyimide composition, typically possess low dielectric constants [152]. Low dielectric constants and low losses are the advantages of PAs. DIW technology further extends these advantages by enabling conformal, lightweight substrates and radome-like housings [153]. The dielectric response of PA systems is governed by several micro- and meso-scale structural parameters [154]. Effective permittivity scales approximately with the solid volume fraction, higher porosity reduces the effective dielectric constant but can increase dispersion if pores and voids produce interfacial polarization [155,156]. Mesopores and micropores (high surface area) increase interfacial polarization (Maxwell-Wagner-Sillars effects) and can elevate dielectric loss at lower frequencies [154,157,158,159]. Polyimide chemistries with polar substituents (e.g., imide N-O, carbonyl dipoles) raise ε’ and dielectric loss, fully aromatic, less polar backbones give lower intrinsic permittivity [160,161]. Dielectric fillers (silica) lower ε’ when they are lower-*k* than PI matrix but raise mechanical stiffness; conductive fillers (rGO, CNT) strongly increase ε’and loss when approaching percolation and contribute to EMI shielding via reflection and absorption. Trapped polar solvents or humidity increase dielectric loss and can shift resonance features [128,162,163,164].

**Figure 9:**
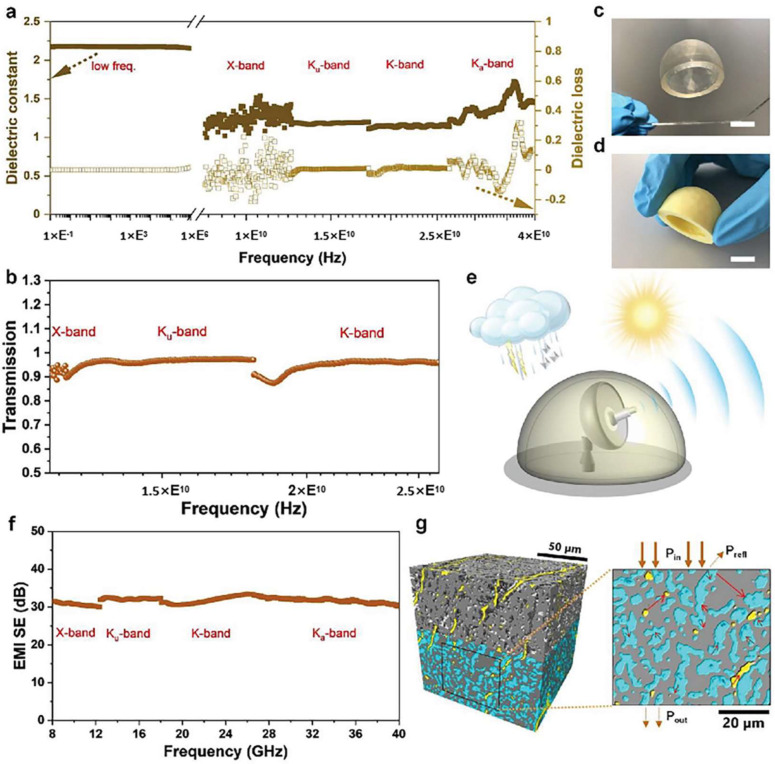
Characterization of the dielectric properties, microwave transmission, and Electromagnetic Interference Shielding Effectiveness (EMI SE) of 3D-printed polyimide/silica composite aerogels. The data include (**a**) the frequency-dependent dielectric constant and loss, (**b**) microwave transmission performance. (**c**,**d**) show the aerogel radome in its as-printed and dried states, respectively (scale bar: 10 mm). (**e**) a conceptual diagram of the composite aerogel radome. (**f**) the EMI SE of carbonized polyimide-silica-rGO composites (2.92 mm thickness). (**g**) 3D rendering with silica aerogel grains (light green) embedded in carbonized polyimide matrix (gray) and rGO (yellow) (100 × 100 × 100 µm^3^, effective voxel size of 162.5 nm); 2D image with arrows to demonstrate the possible shielding mechanism.

Wu et al. fabricated a polyimide–silica aerogel radome structure using mesoporous silica aerogel particles and a polyimide aerogel matrix. The polyimide backbone is derived from BPDA/ODA, which was chosen for its high aromatic content and thermal stability. This yields the high char-forming ability and mechanical robustness necessary for the intended EMI/thermal applications [84]. Figure 9a–d show the dielectric constant, microwave transmission, and photographic images of printed/dried radome specimens, respectively. They achieved dielectric constants and losses in the range of 1.00–1.50 and −0.2–0.2, respectively, in the microwave X-band (8.2–12.4 GHz). At higher frequencies, such as the Ku-band (12.4–18 GHz) and K-band (18–26 GHz), the dielectric constants and losses were in the range of 1.15–1.17 and −0.03–0.02, respectively. Furthermore, Figure 9b presents microwave transmission data for the printed polyimide-silica radome structure, more than 90% of the microwave transmission is detected in the frequency range of 12 to 26 GHz. The high transmittance indicates a low effective dielectric constant and low loss across X-Ku-K bands, which is consistent with a high-porosity matrix and the inclusion of low-index silica aerogel particles that reduce the composite effective permittivity. The bandwidth and nearly flat transmission characteristic indicate both good impedance matching to free space and minimal resonant scattering within the measured frequency range. The radome is undisturbed at radio frequency (RF) and can protect the radar from harsh environments. rGO and CNTs were added to a composite aerogel and then carbonized. This demonstrated a high electromagnetic interference (EMI) shielding effectiveness (SE) of 30–32 dB across an ultra-broadband gigahertz frequency range, encompassing the X-band, Ku-band, K-band, and Ka-band (Figure 9f). The high EMI-specific SE is composed of a conductive rGO-doped carbonized polyimide scaffold surrounding non-conductive silica aerogel particles [135]. The synergistic effects of conductive losses and multiple reflections of the incident electromagnetic wave effectively promote wave attenuation (Figure 9g), with specific SEs (SSEs) of 220–245 dB cm^3^g^−1^ [135]. This electromagnetic wave attenuation is achieved by multiple interface attenuation effects within the carbonized composite material.

**Figure 10:**
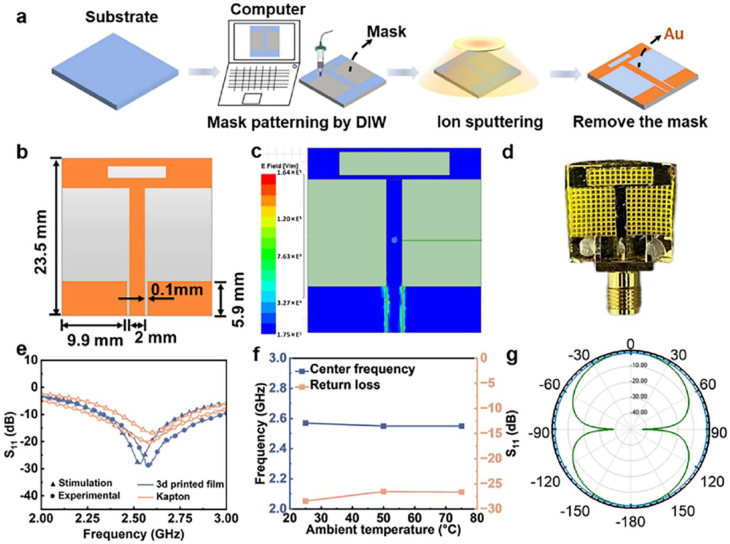
(**a**) Fabrication process of DIW PAs dielectric layer coplanar waveguide antenna. (**b**) Dimensions of the coplanar waveguide antenna and (**c**) Electric field distribution. (**d**) Photograph of the coplanar waveguide antenna. (**e**) Return loss curves of the two antennas. (**f**) Variation in antenna center frequency and return loss with temperature. (**g**) Simulation results.

Li et al. have fabricated PA (prepared by *N*, *N*-Dimethylformamide (DMF), ethanol and soluble polyimide. Soluble polyimide prepared by 4,4-(hexafluoroisopropylidene) diphthalic anhydride (6FDA), 2,2-bis[4-(4-aminophenoxy)phenyl]-hexafluoropropane (BAPOFP) and anhydrous dimethylacetamide (DMAc) mixed with AA and PD) meshes with hierarchical porous structures vis DIW 3D printing. Figure 10a presents the overall morphology of the DIW-printed polyimide aerogel architecture, highlighting its well-defined filament arrangement and high structural fidelity. The dielectric constant of a material is governed by its porosity, meshes with different porosities have distinct dielectric properties [165]. The porosity of the macro-mesh is directly controlled by the filament spacing. The highest overall porosity was obtained when the distance between adjacent printed wires was 2 mm. The resulting material exhibited a porosity of 87%, yielding a minimum dielectric constant of 1.32 and a dielectric loss of 0.005. The combination of non-solvent-induced phase separation and DIW method allowed for the flexible adjustment of the relative dielectric constant [166]. Moreover, the dielectric properties of this grid remained stable in a humid environment. After placing samples with a porosity of 0.60–0.87 in a 75% RH environment for 8 h, the dielectric constant of the grid only increased by 1.4–4.1% compared with the ambient conditions. At different porosities, the grid still had good surface density and moisture resistance without the need for high-temperature post-curing. A DIW 3D-printed recyclable polyimide-silica (RPS) aerogels dielectric film was used as a coplanar waveguide antenna. The antenna structure was designed using a high-frequency structure simulator (Figure 10b). Figure 10c,d further reveal the hierarchical porous network observed at higher magnification, demonstrating uniform pore distribution and continuous skeleton connectivity that together contribute to the material’s low density and favorable mechanical response. The antenna exhibited an omnidirectional radiation field in the x-y plane. The coplanar waveguide antenna is connected to a subminiature A-version (SMA) connector and the resonant frequency is measured to be 2.56 GHz. The resonant frequency is stable between 25 and 75 °C, the amplitude is −28.4 dB, and the antenna bandwidth is 610 MHz, which fully meets the operating frequency requirements of many electronic systems (Figure 10e). The 3D-printed substrate antenna exhibits better performance to the Kapton antenna, as evidenced by its enhanced transmission quality and excellent stability at different temperatures. Figure 10f,g show the functional performance evaluation, where the thermal and mechanical stability of the printed aerogel under operating conditions is visualized and quantified, confirming that the architected structure maintains its integrity and functional reliability without noticeable deformation or degradation [167].

### 3.3. Gas Permeability, Photothermal Conversion, Adsorption and Acoustic

PAs can also serve as promising materials for environmental improvement, such as ammonia absorption and moisture absorption, and seawater purification [83,168,169]. DIW 3D-printed PA can withstand harsh environments and conditions.

**Figure 11:**
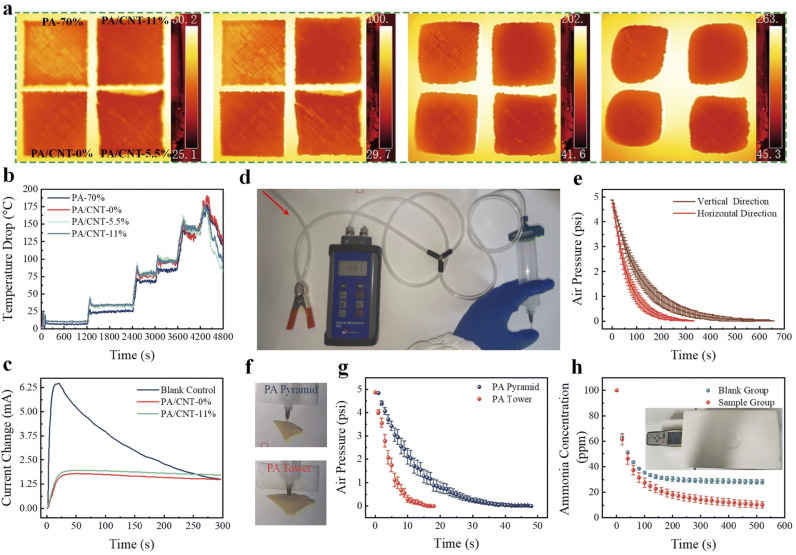
(**a**) Thermal images of DIW PAs at different temperatures. (**b**) Effect of heating stage temperature on PA surface. (**c**) Current versus time curve. (**d**) Simple air permeability test apparatus. (**e**) Air permeability data. (**f**) Optical photographs of the test. (**g**) Air permeability results of DIW PAs. (**h**) Adsorption of ammonia by DIW PAs.

Yang et al. demonstrated excellent air permeability in a 3D-printed PA permeability test device. Figure 11a–c demonstrate the excellent thermal insulation performance of PAs. Figure 11d shows a simplified air permeability testing device. Figure 11e confirms the air permeability of PAs. By varying the stacking ratio, 3D printing can efficiently and reliably create macroscopic periodic structures. Figure 11f,g show that even in thick and complex PA structures, excellent air permeability is maintained. The carboxylic acid and hydroxyl groups present on the multilayer porous framework of 3D-printed PA can serve as acidic sites for ammonia capture, making ammonia adsorption feasible. To monitor the ammonia adsorption, a trace amount of ammonia water was enclosed with the printed structure in a confined space. The concentration dropped to approximately 10 ppm in 520 s, a value lower than the 28 ppm recorded in the control (Figure 11h) [83].

**Figure 12:**
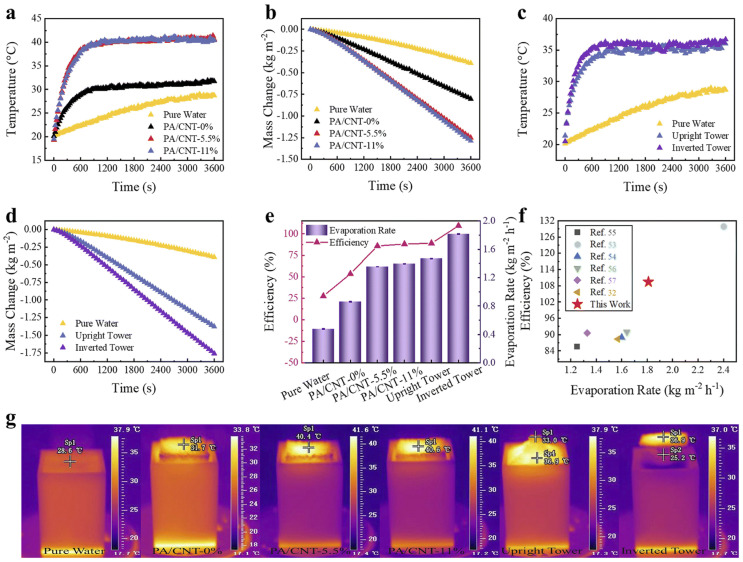
(**a**) Evaporator surface temperature change over time. (**b**) Water quality change caused by evaporator temperature change over time. (**c**) Tower-shaped evaporator surface temperature change over time. (**d**) Water quality change caused by tower-shaped polyamide evaporator temperature change over time. (**e**) Efficiency and evaporation rate of square and tower-shaped evaporators. (**f**) Performance comparison of the evaporator in this study with other reported 3D-printed evaporators. (**g**) Infrared thermogram of the evaporator under thermal equilibrium conditions.

Benefiting from the efficient photothermal conversion provided by carbon nanotubes, 3D-printed PA has shown great potential for solar steam generation (SSG). Adding CNTs to a 3D-printed SSG evaporator significantly improves evaporator efficiency (Figure 12a,b). A comparison of square and tower-shaped evaporators shows that geometry influences evaporator performance. The tower shape is slightly superior to the square evaporator (Figure 12c). The inverted evaporator, due to its concentrated heat and reduced heat loss, achieves a slightly higher evaporation efficiency than the upright evaporator (Figure 12d–f). Infrared thermal imaging in Figure 12g demonstrates the optimal performance of the inverted tower-shaped evaporator, exhibiting faster temperature rise, a slightly higher equilibrium temperature, and greater variation in water mass. The optimized 3D-printed inverted tower-shaped evaporator achieves an excellent evaporation rate of 1.81 kg m^−2^ h^−1^ and an efficiency of 109%. Compared to vertical evaporators, inverted tower evaporators offer superior heat localization and distribution. This effectively reduces heat loss, which is beneficial for steam production. This demonstrates the feasibility of 3D printing to fabricate high-performance SSG evaporators [83].

**Figure 13:**
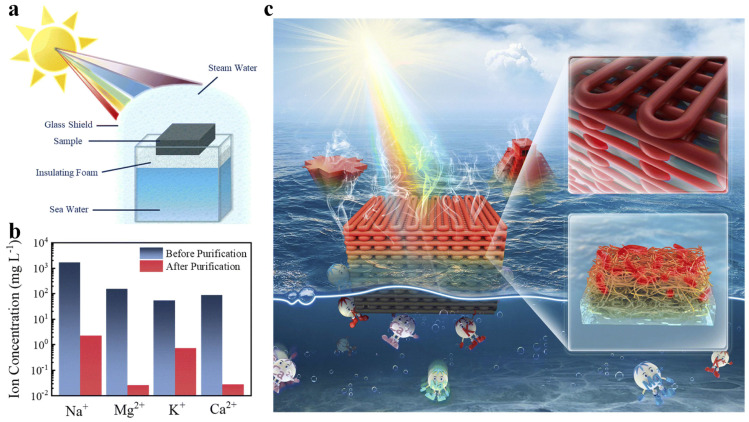
(**a**) Schematic diagram of the apparatus. (**b**) Comparison of metal ion concentration in the desalinated steam water with that in seawater. (**c**) Schematic diagram of the application.

DIW PAs have shown application potential in the field of solar-powered seawater purification because PAs have nanoscale porosity and periodic macroscopic structure, and their adsorption capacity can be enhanced by adding functional fillers [170]. A schematic diagram of the device is shown in Figure 13a. The printed PA evaporator exhibits efficient light-to-heat conversion, expands the contact area with water and light, and optimally localizes heat, effectively reducing heat dissipation and facilitating seawater purification. The concentrations of Na^+^, Mg^2+^, K^+^, and Ca^2+^ were reduced to 2.18, 0.03, 0.70, and 0.03 mg L^−1^, respectively, which is below the recommended values set by the World Health Organization (Figure 13b). Figure 13c shows that the structural design of 3D printed PAs increases the contact area between the evaporator and water and light, effectively reducing heat dissipation and enhancing seawater purification capabilities [83].

The 3D-printed PA exhibits excellent high-frequency sound absorption attributed to the integration of high porosity and large surface area [53]. Compared with conventional gel and foam acoustic materials (e.g., PU foams, silica aerogels, cellulose-based aerogels), polyimide aerogels offer distinct advantages for high-performance and harsh-environment applications. (1) Superior thermo-oxidative stability (useful above 300–400 °C), enabling acoustic mitigation at elevated temperatures where organic foams degrade. (2) A chemically robust imide backbone that preserves mechanical integrity under thermal cycling and mechanical loading, allowing thinner structures and higher specific absorption. (3) Tunable porosity and stiffness via monomer selection and filler addition, which enables impedance matching to air across targeted frequency bands. And (4) compatibility with multifunctional fillers (magnetic, conductive) to broaden absorption mechanisms (visco-thermal, magnetic, and dielectric losses) [14,167,171,172,173]. Limitations include higher material and processing costs and greater post-processing complexity compared to commodity foams [174,175]. PI-based acoustic solutions are most competitive in demanding aerospace, engine-inlet, or high-temperature industrial applications. Air vibrations were generated when sound waves enter the pores of aerogels [172]. The dissipation of sound energy is due to the compression of the air within the small pores, which causes the air to heat up and then attenuate and absorb the sound [176].

**Figure 14:**
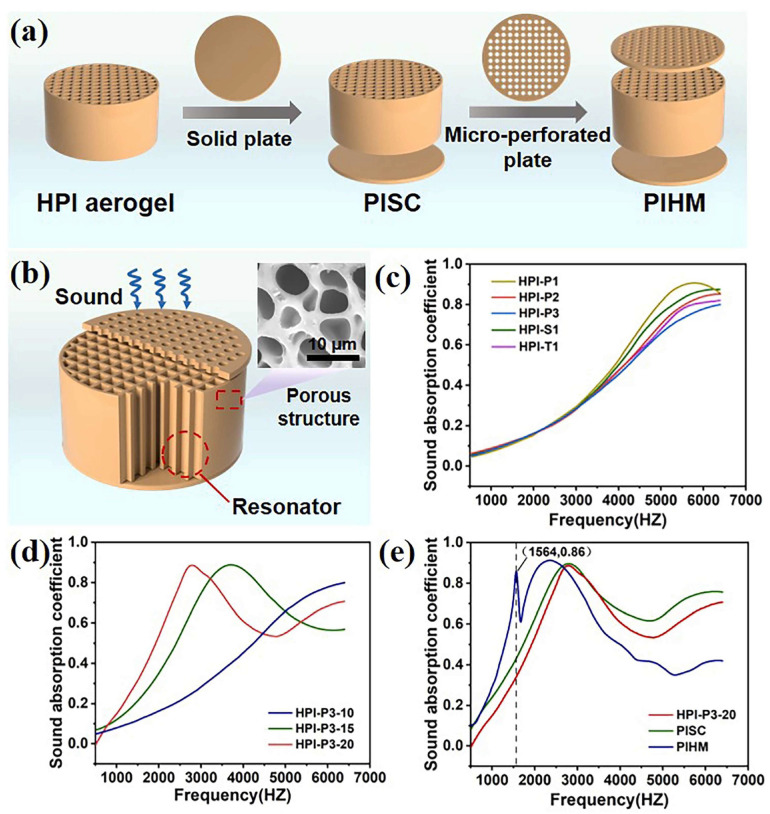
(**a**) Three different sound-absorbing structures. (**b**) PIHM sound-absorbing structure. (**c**) The effect of honeycomb porosity on sound absorption. (**d**) Sound absorption curves for different thicknesses. (**e**) Sound absorption curves for different structures.

High-frequency sound energy is poorly absorbed by lightweight porous PAs. The grid-like honeycomb structure reduces surface reflection, allowing more sound energy to be absorbed and dissipated by the pore walls [53]. Gui et al. assembled a 3 mm thick solid circular base plate printed by DIW 3D printing with a single honeycomb channel building block to form a HPI aerogel semi-enclosed cavity (PISC) (Figure 14a). A parallel microporous plate was added to form a multiple Helmholtz resonator (PIHM) (A Helmholtz resonator consists of a cavity coupled to the external medium via a neck, resonance arises when mass inertia in the neck couples to compressibility of the cavity volume, producing a strong absorptive peak at a characteristic frequency. Hierarchically printed PAs can host many such cavity–neck motifs at different length scales; these produce multiple, spectrally separated Helmholtz resonances that overlap to form a broad absorption band) (Figure 14b). Increasing the porosity from 60% to 75% and the honeycomb side length from 0.5 mm to 2 mm reduced the vibration friction generated by sound waves entering the honeycomb channel, converting the sound energy into heat and subsequently decreasing the sound absorption coefficient (Figure 14c). The discrete absorption peaks observed in Figure 14c–e arise from different, coexisting dissipation mechanisms. Low-frequency peaks are primarily due to macroscopic structural resonances, including PIHMs formed by the printed lattice cavities and channels. The resonance frequency is controlled by cavity volume, neck area, and effective neck length. Viscous dissipation associated with air flow in mesopores and the frictional interaction between air and pore walls (viscous boundary-layer losses) produce mid-band attenuation. Pore throat constriction and tortuosity intensify these losses. At higher frequencies, intrinsic viscoelastic damping of the polyimide network and interfacial polarization at filler–matrix boundaries increase loss tangent and broaden the absorption spectrum. As the thickness increases, the sound absorption performance in the low-frequency range gradually deteriorates. PISC structure achieves an absorption coefficient of approximately 0.9 at 2800 Hz. Compared with PISC, PIHM shifts the absorption peak at 2800 Hz to the left, slightly increasing the sound absorption coefficient. A new absorption peak appears at 1564 Hz, with a sound absorption coefficient of 0.86 [53]. This demonstrates the feasibility of DIW 3D-printed PAs structures for acoustic applications.

## 4. Summary and Outlook

DIW has established itself as a versatile and practical manufacturing route for architecting PAs, enabling precise hierarchical control over macro- and meso-porosity, the fabrication of complex conformal shapes, and the integration of multimaterial structures-capabilities that are challenging or unattainable with conventional mold-based sol–gel processing. This review has systematically synthesized current ink design strategies, compared drying and imidization pathways, and summarized application-driven performance in areas spanning thermal insulation, dielectric substrates, EMI shielding, photothermal conversion, and acoustic metamaterials.

The successful DIW of PAs hinges on a specific rheological profile that balances extrudability with shape fidelity, coupling pronounced shear-thinning with rapid thixotropic recovery and a yield stress typically between 10–10^3^ Pa. The resulting material properties are co-determined by porosity, pore-size distribution, polymer backbone polarity, and filler type, while the post-processing steps–specifically the drying route and imidization schedule-exert a critical influence on volumetric shrinkage, microstructural integrity, and ultimate thermal stability. Supercritical CO_2_ drying emerges as being particularly effective for porosity preservation, albeit at elevated cost and complexity.

Addressing several critical challenges is essential for future advancements in the field. A primary research direction involves resolving the fundamental printability–porosity trade-off through the development of predictive rheological models and quantitative process maps. Concurrently, advanced shrinkage mitigation strategies–such as graded curing, localized imidization, and hybrid drying—are needed to achieve shape fidelity with minimal deformation. The transition to industrial relevance further demands a concerted focus on sustainable and scalable processing, including solvent substitution, closed-loop recovery, and continuous production systems. Finally, establishing standardized, application-grade reliability testing protocols and pursuing recyclable PI chemistries will be crucial for validating long-term performance in demanding environments and promoting material circularity. Through synergistic advances in predictive modeling, low-impact processing, and rigorous validation, DIW of polyimide aerogels can transition from academic demonstration to functional, engineered solutions for thermal protection, radome substrates, and harsh-environment applications. The consolidated analysis and prioritized research directions presented here are intended to accelerate this transition.

## Figures and Tables

**Table 1:** Examples of PA inks suitable for DIW 3D printing.

Composition of Aerogel		Drying Method and Post-Treatment After Drying	Applications	Ref.
Main Raw Material	Additive			
ODA and triethylamine (TEA), PMDA	CNCs	Freeze drying, thermal imidization	Thermal insulation	[73]
	BC			[134]
ODA, 4,4′-Diphenoxydianiline (ODPA), 2-Dimethylamino ethanol (DMAE), TEA	-	Freezing-extraction, chemical imidization and thermal treatment	Aerospace applications	[88]
2,2′-Dimethylbenzidine (DMBZ), 4,4′-hexafluoroisopropylidene di(phthalic anhydride) (6FDA), PMDA, 1,3,5-Triaminophenoxybenzene (TAB), TEA	-	Drying under reduced pressure	Battery thermal mitigation	[74]
2,2′-dimethyl-[1,1′-biphenyl]-4,4′-diamine (DMBZ), Pyridine (PD), benzophenone-3,3′, 4,4′-tetracarboxylic dianhydride (BTDA)	CNTs	One-step chemical imidization	Thermal insulation, gas permeability and light absorption	[83]
ODA, DMBZ, 3,3′,4,4′-biphenyl tetracar boxylic dianhydride (BPDA)	SA	Chemical imidization and supercritical CO_2_ drying	Electromagnetic interference shielding and thermal management	[135]
BPDA, ODA, NMP	SA	Chemical imidization and supercritical CO_2_ drying	Thermal insulation	[136]
BPDA, ODA, NMP, TEA	-	Freeze drying, thermal imidization	Sound absorbing metamaterials	[53]
2,2-Bis [4-(4-aminophenoxy)phenyl]propane (BAPP), 2,2-bis [4-(3,4-dicarboxyphenoxy) phenyl]propanedianhydride (BPADA)	SA	Freeze drying, thermal imidization	Shape memory and thermal insulation materials	[84]

**Table 2:** Rheological properties of inks.

Composition of Aerogel	Viscosity (Pa·s)	Storage Modulus G′ (Pa)	Loss Modulus G″ (Pa)	Yield Stress(Pa)	Ref.
PA/CNCs nanocomposites	228–18,134	~10^3^–10^4^	~300–600	>200	[73]
PAs	6.03 ± 1.52–9.93 ± 3.75	<10^3^	<10^3^	-	[74]
PAs	-	<7 × 10^4^	-	-	[135]
PAs	<1.4 × 10^4^	4.6 × 10^2^–1.4 × 10^5^	-	-	[136]
PAs	20–328	~1 × 10^3^–2 × 10^3^	~4 × 10^2^–5 × 10^2^	-	[53]
PAs	4 × 10^1^–6 × 10^7^	~1 × 10^1^–9 × 10^3^	~1 × 10^1^–7 × 10^3^	-	[86]
PAs	-	-	-	-	[85]
PAs	-	-	-	-	[53]
PAs	-	4 × 10^4^	-	2700	[51]
PAs	-	2.9 × 10^3^–1.34 × 10^4^	-	433–3594	[84]
PAs	-	5.01 × 10^2^–1.82 × 10^3^	-	27–46	[87]

## Data Availability

No new data were created or analyzed in this study.
